# Non-Adiabatic Excited-State Time-Dependent *GW* (TD*GW*) Molecular Dynamics Simulation of Nickel-Atom Aided Photolysis of Methane to Produce a Hydrogen Molecule

**DOI:** 10.3390/nano14221775

**Published:** 2024-11-05

**Authors:** Aaditya Manjanath, Ryoji Sahara, Yoshiyuki Kawazoe, Kaoru Ohno

**Affiliations:** 1 Research Center for Structural Materials, National Institute for Materials Science (NIMS), 1-2-1 Sengen, Tsukuba 305-0047, Ibaraki, Japan; manjanath.aaditya@nims.go.jp (A.M.); sahara.ryoji@nims.go.jp (R.S.); 2 New Industry Creation Hatchery Center, Tohoku University, 6-6-4 Aramaki Aza Aoba, Aoba-ku, Sendai 980-8579, Miyagi, Japan; kawazoe@e-workshop.co.jp; 3 School of Physics, Institute of Science, Suranaree University of Technology, 111 University Avenue, Nakhon Ratchasima 30000, Thailand; 4 Physics and Nanotechnoloy, SRM Institute of Science and Technology, Tamil Nadu, Kattankurathur 603203, India; 5 Department of Physics, Graduate School of Engineering, Yokohama National University (YNU), 79-5 Tokiwadai, Hodogaya-ku, Yokohama 240-8501, Kanagawa, Japan

**Keywords:** quasiparticle, Koopmans’ theorem, TDDFT, GW approximation, Ni atom, CH_4_, chemical reaction, photochemistry, surface hopping

## Abstract

Methane photolysis is a very important initiation reaction from the perspective of hydrogen production for alternative energy applications. In our recent work, we demonstrated using our recently developed novel method, non-adiabatic excited-state time-dependent GW (TDGW) molecular dynamics (MD), how the decomposition reaction of methane into a methyl radical and a hydrogen atom was captured accurately via the time-tracing of all quasiparticle levels. However, this process requires a large amount of photoabsorption energy (PAE ∼10.2 eV). Moreover, only one hydrogen atom is produced via a single photon absorption. Transition metal atoms can be used as agents for photochemical reactions, to reduce this optical gap and facilitate an easier pathway for hydrogen production. Here, we explore the photolysis of methane in the presence of a Ni atom by employing TDGW-MD. We show two possibilities for hydrogen-atom ejection with respect to the location of the Ni atom, towards the Ni side or away from it. We demonstrate that only the H ejection away from the Ni side facilitates the formation of a hydrogen molecule with the quasiparticle level corresponding to it having an energy close to the negative ionization potential of an isolated H_2_ molecule. This is achieved at a PAE of 8.4 eV which is lower compared to that of pristine methane. The results obtained in this work are an encouraging step towards transition metal-mediated hydrogen production via photolysis of hydrocarbons.

## 1. Introduction

Methane is a very important fuel gas as (a) it is a main constituent of liquefied natural gas (LNG) [1], which is useful for long-distance transport, (b) it is the main component of biofuel or biogas [2], making it a source of clean energy, and (c) it is useful as a direct coolant in jet engines. In addition, methane serves as a precursor gas for hydrogen production. H_2_ is considered the “fuel of the future” because it produces three times the amount of energy (39.4 kWh ${\mathrm{kg}}^{-1}$) compared to other fuels such as liquid hydrocarbons (13.1 kWh ${\mathrm{kg}}^{-1}$) [3]. It has been reported extensively that the endothermic decomposition of methane leads to the production of hydrogen. Cracking [4] (heating of methane in the absence of air), photolysis [5,6,7,8,9] (photoexcitation of methane), and steam reforming [10,11] (reaction of methane with steam), as well as thermocatalytic decomposition [12,13,14,15,16] and solar-aided decomposition [17,18,19,20], both of which produce hydrogen along with “carbon black” or nano-sized carbon clusters, without emitting greenhouse gases, are the most commonly employed processes for this.

From the perspective of hydrogen production, it is interesting and important to investigate the photochemical reactions of hydrocarbons. The computational approach is more favorable than the typical experimental approach when investigating ultrafast reactions such as photolysis. However, density functional theory (DFT) [21] is, in principle, a ground-state theory and cannot be applied to any photoexcited state. Time-dependent density functional theory (TDDFT) [22] relying on adiabatic local density approximation (ALDA) [23] has been the standard approach to investigate the excited-state (ES) dynamics; however, it faces a longstanding difficulty of ALDA not being applicable to the time-dependent (TD) molecular dynamics (MD) for an initially excited state (ES). We have recently overcome this difficulty [24] by employing extended quasiparticle theory (EQPT) [25,26], in which the full correspondence is achieved between the ES surfaces and corresponding total energies, with satisfying extended Koopmans’ theorem [27,28]. In EQPT, each quasiparticle (QP) level is related to a total energy difference. The QP energies of an occupied level ($\varepsilon_{i}^{\mathrm{QP}}$) and an unoccupied level ($\varepsilon_{a}^{\mathrm{QP}}$) are defined, respectively, as

(1a) $$\begin{array}{cc} \varepsilon_{i}^{\mathrm{QP}} & =E_{\mathrm{ref}}^{\left(N\right)}-E_{i\to \mathrm{vac}}^{\left(N-1\right)}, \end{array}$$

(1b) $$\begin{array}{cc} \varepsilon_{a}^{\mathrm{QP}} & =E_{\mathrm{vac}\to a}^{\left(N+1\right)}-E_{\mathrm{ref}}^{\left(N\right)}, \end{array}$$

where $E_{\mathrm{ref}}^{\left(N\right)}$, $E_{i\to \mathrm{vac}}^{\left(N-1\right)}$, and $E_{\mathrm{vac}\to a}^{\left(N+1\right)}$ are the total energies of the reference *N*-electron system, the (N−1)-electron system formed by removing an electron from the *i*th occupied level to the vacuum level (vac), and the (N+1)-electron system formed by adding an electron at the *a*th unoccupied level from vac. Here, we emphasize that the reference *N*-electron system is not necessarily the ground state (GS), but can be any of the excited eigenstates. The QP energies $\varepsilon_{i}^{\mathrm{QP}}$ and $\varepsilon_{a}^{\mathrm{QP}}$ in Equation (1) can be referred to as ‘negative ionization potentials’ and ‘negative electron affinities’, respectively, and have a direct correspondence with the observations from photoemission/inverse photoemission experiments. The GW approximation (GWA) is in full conformity with EQPT. We have applied EQPT within the GWA to the mixed quantum-classical Ehrenfest dynamics with surface hopping (SH) and developed the NA-ES-TDGW (TDGW) MD method [24]. The merit of this method is that we can trace all the QP energies as well as the QP wavefunctions, which we will refer to as “charge densities”, during the simulation. Using this method, we have successfully investigated methane photolysis CH_4_ $\overset{\hbar \omega }{\to }$ CH_3_· + H at the lowest photoexcited state [24], which is considered as the initiation reaction of a complex multi-step process of a variety of methane combustion and hydrogen production reactions.

Nevertheless, the photolysis of methane requires a large photoexcitation energy. In this regard, transition metal atoms can pave the way for reducing such large optical gaps and facilitate an easier pathway for hydrogen production. Here, we focus on the effect of a transition atom in this reaction. The aim of the present study is to unravel new reaction pathways in nickel atom-mediated methane photolysis. To the best of our knowledge, there has been no direct molecular dynamics study to search the reaction pathway of ${\mathrm{CH}}_{4}$ in the presence of a transition metal atom at any of the photoexcited states, although there have been several computational studies involving systems with transition metal atoms. One such study is the TDDFT molecular dynamics investigation of the hydrogen spillover process via Ni dimers by Sahara et al. [29]. They showed that a hydrogen molecule can be dissociated into two hydrogen atoms near the Ni dimer by a small excitation energy. Another is a DFT study on the potential energy surfaces of a CH_4_ molecule with a Ni atom by Burghgraef et al. [30] or with an Fe atom by Sun et al. [31]. From their results, reactions such as CH_4_ + M → CH_3_MH (M = Ni or Fe) can be expected. Concerning the late-stage dynamics of the thermocatalytic decomposition of methane, there is a DFT study on the potential energy surface of a ${\mathrm{Ni}}_{55}$ cluster with an open (semi-capped) carbon nanotube by Wang et al. [32]. Although the earlier stage of methane decomposition was not considered in their work, the mechanism of the catalytic behavior of the Ni cluster was clarified.

The presence of a Ni atom breaks the tetrahedral symmetry of CH_4_, rendering a non-unique choice of the photoexcited state. The highest occupied molecular orbital (HOMO) of this system is no more that of the methane fragment but is now one of the highest occupied Ni 3d levels. Moreover, the lowest unoccupied molecular orbital (LUMO) of this system is no more than the negative electron affinity level of pure CH_4_ above the vacuum level but is the lowest unoccupied Ni 3d level, which is below the vacuum level. This means that there are several possible reaction pathways depending on the choice of the photoexcited excited state, for which the excitation energy is lower than 10.2 eV [5,6,7] that is needed in the photolysis of the pristine methane case. In the course of our study, we show that there are at least two possible pathways of methane decomposition. In one case, hydrogen atoms on the side opposite Ni are ejected from the methane molecule, while in the other case, hydrogen atoms facing the Ni atom are ejected from the methane molecule. We present the results of the two different pathways in detail in Section 3.

## 2. Method

### 2.1. Time-Dependent QP Equation

The wavepackets $\phi_{\lambda }\left(r;R\left(t\right),t\right)$, where r, $R\left(t\right)=\{R_{I}\left(t\right)\}$, and *t* represent the position of the QP, a set of nuclear coordinates (*I* - atomic index), and time, respectively, satisfy the time-dependent QP (TDQP) equation [24]

(2) $$\begin{array}{c} i\frac{\partial }{\partial t}\phi_{\lambda }\left(r;R\left(t\right),t\right)=H_{\mathrm{mixed}}^{\mathrm{QP}}\left(r;R\left(t\right)\right)\phi_{\lambda }\left(r;R\left(t\right),t\right), \end{array}$$

which is similar to the time-dependent Kohn–Sham equation [22]. Here, $H_{\mathrm{mixed}}^{\mathrm{QP}}\left(r;R\left(t\right)\right)$ denotes the QP (GW) Hamiltonian for the mixed state constructed by these wavepackets [24]. We introduce the QP wavefunctions $\varphi_{n}^{\mathrm{QP}}\left(r;R\left(t\right)\right)$ and the QP energies $\varepsilon_{n}^{\mathrm{QP}}\left(R\left(t\right)\right)$ [n=i (occupied) or *a* (unoccupied), QP level indices], which satisfy the eigenvalue equation

(3) $$\begin{array}{c} H_{\mathrm{mixed}}^{\mathrm{QP}}\left(r;R\left(t\right)\right)\varphi_{n}^{\mathrm{QP}}\left(r;R\left(t\right)\right)=\varepsilon_{n}^{\mathrm{QP}}\left(R\left(t\right)\right)\varphi_{n}^{\mathrm{QP}}\left(r;R\left(t\right)\right) \end{array}$$

during the simulation. Then, using the spectral method [24,29,33], we can expand the wavepackets $\phi_{\lambda }\left(r;R\left(t\right),t\right)$ as

(4) $$\begin{array}{cc} \phi_{\lambda }\left(r;R\left(t\right),t\right)\; & =\sum_{n}\varphi_{n}^{\mathrm{QP}}\left(r;R\left(t\right)\right)c_{n\lambda }\left(t\right) \end{array}$$

with the expansion coefficients

(5) $$\begin{array}{c} c_{n\lambda }\left(t\right)=\langle \;\varphi_{n}^{\mathrm{QP}}\left(R\left(t\right)\right)\;|\;\phi_{\lambda }\left(R\left(t\right),t\right)\;\rangle . \end{array}$$

The TDQP Equation (2) can be numerically solved by propagating the wavepackets in small timesteps Δt. The wavepackets $\phi_{\lambda }\left(r;R\left(t+\Delta t\right),t+\Delta t\right)$ at a slightly later time t+Δt with a small time interval Δt can be expressed as

(6) $$\begin{array}{cc} \phi_{\lambda }\left(r;R\left(t+\Delta t\right),t+\Delta t\right)\; & =\exp\left[-i\int_{t}^{t+\Delta t}H_{\mathrm{mixed}}^{\mathrm{QP}}\left(r;R\left(t\right)\right)dt\right]\;\phi_{\lambda }\left(r;R\left(t\right),t\right)\\ \; & \approx \sum_{n}\exp\left[-i\varepsilon_{n}^{\mathrm{QP}}\left(R\left(t+\Delta t\right)\right)\right]\;\varphi_{n}^{\mathrm{QP}}\left(r;R\left(t+\Delta t\right)\right)c_{n\lambda }\left(t+\Delta t\right) \end{array}$$

with

(7) $$\begin{array}{cc} c_{n\lambda }\left(t+\Delta t\right)\; & =\langle \;\varphi_{n}^{\mathrm{QP}}\left(R\left(t+\Delta t\right)\right)\;|\;\phi_{\lambda }\left(R\left(t\right),t\right)\;\rangle \\ \; & \approx \langle \;\varphi_{n}^{\mathrm{QP}}\left(R\left(t\right)\right)\;|\;\phi_{\lambda }\left(R\left(t\right),t\right)\;\rangle +\Delta t\frac{\partial }{\partial t^{\prime }}{\langle \;\varphi_{n}^{\mathrm{QP}}\left(R\left(t^{\prime }\right)\right)\;|\;\phi_{\lambda }\left(R\left(t\right),t\right)\;\rangle }_{t^{\prime }=t}\\ \; & =c_{n\lambda }\left(t\right)-\Delta t\sum_{m}c_{m\lambda }\left(t\right)\sum_{I}{\dot{R}}_{I}\left(t\right)\langle \;\varphi_{n}^{\mathrm{QP}}\left(R\left(t\right)\right)\;|\;\nabla_{R_{I}}\;|\;\varphi_{m}^{\mathrm{QP}}\left(R\left(t\right)\right)\;\rangle . \end{array}$$

In the last equality of Equation (7), we used Equation (4) again. The second term of the rightmost expression in Equation (7) is proportional to the nuclear velocity ${\dot{R}}_{I}\left(t\right)=dR_{I}\left(t\right)/dt$ and represents a non-adiabatic interaction [34,35]. The matrix elements $d_{nm}=\langle \;\varphi_{n}^{\mathrm{QP}}\left(R\left(t\right)\right)\;|\;\nabla_{R_{I}}\;|\;\varphi_{m}^{\mathrm{QP}}\left(R\left(t\right)\right)\;\rangle$ represent the non-adiabatic coupling vectors.

### 2.2. One-Shot GW Within TDQP

We apply this formalism to the one-shot GW approach [36], which is the simplest and most reliable approach in the GWA. In the one-shot GW approach, the QP wavefunctions are replaced by Kohn–Sham orbitals in the local density approximation (LDA) [36]

(8) $$\begin{array}{c} \varphi_{n}^{\mathrm{QP}}\left(r;R\left(t\right)\right)\approx \varphi_{n}^{\mathrm{LDA}}\left(r;R\left(t\right)\right), \end{array}$$

and the QP energy eigenvalues $\varphi_{n}^{\mathrm{QP}}\left(r;R\left(t\right)\right)$ are calculated from the LDA eigenvalues $\varphi_{n}^{\mathrm{LDA}}\left(r;R\left(t\right)\right)$ and the exchange-correlation potential $\mu_{\mathrm{xc}}^{\mathrm{LDA}}\left(r\right)$ as

(9) $$\begin{array}{c} \varepsilon_{n}^{\mathrm{QP}}\left(R\left(t\right)\right)\approx \varepsilon_{n}^{\mathrm{LDA}}\left(R\left(t\right)\right)+\langle \varphi_{n}^{\mathrm{LDA}}\left(R\left(t\right)\right)|\left[\Sigma_{\mathrm{xc}}\left(\varepsilon_{n}^{\mathrm{QP}}\left(R\left(t\right)\right)\right)-\mu_{\mathrm{xc}}^{\mathrm{LDA}}\right]|\varphi_{n}^{\mathrm{LDA}}\left(R\left(t\right)\right)\rangle , \end{array}$$

where $\Sigma_{\mathrm{xc}}\left(\varepsilon_{n}^{\mathrm{QP}}\left(R\left(t\right)\right)\right)$ is the exchange-correlation part of the self-energy, which does not include the Hartree term. The self-energy $\Sigma_{\mathrm{xc}}\left(\varepsilon_{n}^{\mathrm{QP}}\left(R\left(t\right)\right)\right)$ in Equation (9) explicitly depends on the resulting QP energy $\varepsilon_{n}^{\mathrm{QP}}\left(R\left(t\right)\right)$. The QP energy obtained in a previous time step can be used as the argument for self-energy in the present time step. Therefore, the renormalization procedure using the *Z* factor [36] is not required in the present time-dependent approach. The usage of the QP energies $\varepsilon_{n}^{\mathrm{QP}}\left(R\left(t\right)\right)$, which are obtained by Equation (9) in Equation (6), is the distinguishing feature of the TDGW approach. Except for this difference, everything else remains the same as in the TDDFT dynamics formulation. The Newtonian equation of motion for NA-ES-TDGW-MD is approximated as

(10) $$\begin{array}{cc} M_{I}\frac{d^{2}R_{I}\left(t\right)}{dt^{2}} & =-\nabla_{R_{I}}E_{\mathrm{ref}}^{\left(N\right)}\left(R\left(t\right),t\right)\\ \; & \approx -\nabla_{R_{I}}E_{\mathrm{LDA}}^{\left(N\right)}\left(R\left(t\right),t\right), \end{array}$$

where $E_{\mathrm{ref}}^{\left(N\right)}\left(R\left(t\right),t\right)$ and $E_{\mathrm{LDA}}^{\left(N\right)}\left(R\left(t\right),t\right)$ denote the GW total energy and the approximate LDA total energy, respectively, of an *N*-electron reference state. Although the exchange-correlation contribution to the total energy is approximated by its LDA form, the wavepackets used for TD charge densities are updated by Equation (6). Therefore, $E_{\mathrm{LDA}}^{\left(N\right)}\left(R\left(t\right),t\right)$ is not the typical LDA total energy, but it includes the QP effects via the QP Hamiltonian $H_{\mathrm{mixed}}^{\mathrm{QP}}\left(r;R\left(t\right)\right)$.

### 2.3. Ab Initio Cloning in NA-ES-TDGW-MD

In our approach, the nuclear trajectory evolves as the gradient of the average potential generated by the electrons, which is a mean-field approximation in the Ehrenfest framework. In this mean-field approximation, the correlation (also known as quantum coherence) between the electron “motion” and nuclear trajectory is neglected. This suffers from the problem of strong non-adiabatic mixing particularly when two Born–Oppenheimer (BO) surfaces cross or approach each other. A proper description of such correlations requires a distinct classical trajectory for each electronic state, which is provided by an SH strategy, such as that proposed by Tully [37,38] or Makhov et al. [35]. In this work, we adopt the SH strategy proposed by Makhov et al. [35] named ab initio (multiple) cloning in our dynamics formalism, although our approach does not include multiple trajectory basis functions.

A quantity that is used as a measure of quantum (de)coherence, i.e., hopping from a mixed surface (with index λ) to a pure BO surface (with index *n*) is called the “breaking force” $F_{\lambda \to n}^{\mathrm{br}}$. The indices λ and *n* typically indicate the newly occupied/empty QP level (OCC1/EMP1; see Section 3.1) in the QP representation. The breaking force is defined as

(11) $$\begin{array}{c} F_{\lambda \to n}^{\mathrm{br}}=\left(1-\right|c_{n\lambda }\left(t\right){|}^{2})\Delta F_{n\lambda }\left(t\right), \end{array}$$

where $c_{n\lambda }\left(t\right)$ are the coefficients in the expansion of the wavepacket as defined in Equation (5) or (7) and $\Delta F_{n\lambda }\left(t\right)$ is the deviation of the force felt by the nuclei on a pure BO surface ($-\nabla_{R_{I}}E_{n}^{\left(N\right)}\left(R\left(t\right),t\right)$) from the mean-field force in Equation (10) ($\nabla_{R_{I}}E_{\lambda }^{\left(N\right)}\left(R\left(t\right),t\right)$):

(12) $$\begin{array}{c} \Delta F_{n\lambda }\left(t\right)=\nabla_{R_{I}}E_{\lambda }^{\left(N\right)}\left(R\left(t\right),t\right)-\nabla_{R_{I}}E_{n}^{\left(N\right)}\left(R\left(t\right),t\right). \end{array}$$

The condition for a hop (or “clone” in Ref. [35]) is that the breaking acceleration $a_{\lambda \to n}^{\mathrm{br}}$ is greater than a pre-decided threshold $\delta_{\mathrm{clone}}$,

(13) $$\begin{array}{c} a_{\lambda \to n}^{\mathrm{br}}=\left|M^{-1}F_{\lambda \to n}^{\mathrm{br}}\right|>\delta_{\mathrm{clone}}. \end{array}$$

In this work, we define $\delta_{\mathrm{clone}}=3\times 10^{-6}$ a.u. for exploring the surface hop time as in our previous study [24].

### 2.4. Computational Details

Since the eigenstates span the complete Hilbert space, we use the all-electron mixed-basis approach [24,29,33,39,40] implemented in our home-grown *ab initio* package named Tohoku mixed basis orbitals (TOMBO) [41], in which the one-electron orbitals are expressed by both plane waves (PWs) and atomic orbitals (AOs). We use a simple cubic unit cell of 12Å, where the Coulomb interaction is spherically cut to avoid interactions with adjacent unit cells. The 14147 PWs corresponding to the 17.3 Ry cutoff energy and minimal number of numerical AOs have finite values only within each non-overlapping atomic sphere. AOs are smoothly truncated by subtracting a smooth quadratic function, which has the same amplitude and derivative at the atomic sphere surface [41]. This quadratic function smoothly connecting to the tail of the true orbital can be successfully described by a linear combination of PWs. The cutoff energies corresponding to 69 Ry and 7.7 Ry are set, respectively, for the exchange and correlation parts of the self-energy. 190 levels are used in the spectral decomposition [24] and in the calculation of the correlation part of the self-energy. The generalized plasmon pole model [36] is used to avoid frequency integration. We performed tests to ensure that all these parameters are sufficient for obtaining good convergence of results. However, we increased the cutoff energies to 44 Ry (for PWs), 111 Ry (for exchange), and 11 Ry (for correlation) as well as the number of levels to 3500 when a separate one-shot GW calculation was performed.

The precursor to performing a TDGW molecular dynamics simulation is to obtain converged electronic states at a given electron configuration within the LDA. Once convergence is achieved, the dynamics simulation is performed by updating the wavepackets stepwise in Δt=0.01 fs intervals, where the Hamiltonian is expected to change very slightly. The wavepackets are the same as the QP (Kohn–Sham) eigenfunctions for the ES reference at t=0. We perform 20 sub-loops of electronic state updates after every update of the atomic positions, to ensure that the total energy is conserved. The GW calculation is performed only during the final 20th sub-loop, and the QP energies and atomic positions are updated subsequently.

## 3. Results

**Figure 1:**
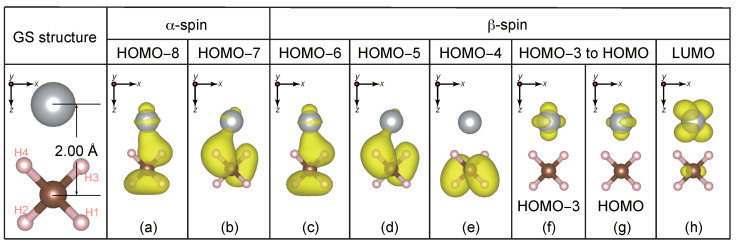
Initial geometry (leftmost panel) and (**a**–**h**) charge densities of the relevant α- and β-spin QP levels for the GS reference. The isosurface for the charge density plots was set at $1\times 10^{-8}$ electrons/${Å}^{3}$. The directions of viewing are indicated for reference.

The initial geometry of the CH_4_ + Ni system is shown in the leftmost panel in Figure 1. The Ni atom is placed at a C−Ni bond distance of 2.00Å, which is the stable distance for carbon chemisorption on a Ni(111) surface [42]. Two H atoms (H1 and H2) are away from the Ni atom while the other two (H3 and H4) face the Ni atom. We impose symmetry breaking of the initial geometry of CH_4_ by slightly elongating one of the C−H bonds facing the Ni atom (H4) by 0.05Å. The CH_4_ + Ni system has a triplet ground state with 20α-spin (↑-spin) and 18β-spin (↓-spin) electrons. The charge density distribution of each QP level for the GS reference of this geometry is depicted in Figure 1. Here, and hereafter, the charge density implies the absolute value of the QP wavefunction-squared irrespective of its occupancy.

From a single-shot GW calculation for the GS reference, the QP energies are obtained as presented in Table 1. Since the 14th level, which corresponds to the HOMO of the methane fragment (indicated by ‘*’ in Table 1), is slightly shallower for β-spin than that for α-spin, we chose the β-spin for electron excitations to the LUMO. The QP energies of the 14th HOMO−$4_{\beta }$ (HOMO of CH_4_), 13th HOMO−$5_{\beta }$ (HOMO−1 of CH_4_), and ${\mathrm{LUMO}}_{\beta }$ levels are, respectively, $\varepsilon_{\mathrm{HOMO}-4_{\beta }}=-14.9$ eV, $\varepsilon_{\mathrm{HOMO}-5_{\beta }}=-15.1$ eV, and $\varepsilon_{{\mathrm{LUMO}}_{\beta }}=-1.0$ eV. Therefore, the ionization potential (IP) of the methane fragment at the GS of the CH_4_ + Ni system, i.e., the energy required to remove one electron from the 14th HOMO−$4_{\beta }$ level, is $-\varepsilon_{\mathrm{HOMO}-4_{\beta }}=14.9$ eV which is higher than that of pristine methane (GW: 13.7±0.5 eV [24] and experiments: 12.6–14.8 eV [43,44,45]), while the electron affinity (EA) of the CH_4_ + Ni system at the GS, i.e., the energy obtained by adding one electron to the 19th ${\mathrm{LUMO}}_{\beta }$ level, is $-\varepsilon_{{\mathrm{LUMO}}_{\beta }}=1.0$ eV. The higher IP and positive EA values in CH_4_ + Ni are attributed to the presence of the Ni *d* orbitals.

The slightly distorted CH_4_ still has a nearly two-fold degenerate ${\mathrm{HOMO}}_{\beta }$ (13th and 14th β-spin levels of the CH_4_ + Ni system), whose QP wavefunctions exhibit two different orientations with respect to the Ni atom, see Figure 1d,e. Therefore, the trajectory differs depending on which level is excited. Here, we show two significantly different trajectories when an electron is excited from the 13th/14th β-spin level to the 19th empty (LUMO) level. The 13th→19th level excitation involves the movement of the two H atoms facing Ni (H3 and H4 in Figure 1) while the 14th→19th level excitation involves the movement of the two H atoms away from Ni (H1 and H2 in Figure 1). We first present the latter case before presenting the former.

### 3.1. H Ejection Opposite to the Ni Side

In this case, we excite an electron from the slightly shallower ${\mathrm{HOMO}}_{\beta }$ level of CH_4_, which is the 14th HOMO−$4_{\beta }$ level of the CH_4_ + Ni system, to the empty Ni 3*d* level, which is the 19th ${\mathrm{LUMO}}_{\beta }$ level. Now, the original HOMO−$4_{\beta }$ is called “${\mathrm{EMP1}}_{\beta }$” and the original ${\mathrm{LUMO}}_{\beta }$, “${\mathrm{OCC1}}_{\beta }$”. The labels such as OCC${\#}_{\alpha /\beta }$ and EMP${\#}_{\alpha /\beta }$ with #=1, 2 ,… indicate the lower occupied and higher empty levels, respectively. This nomenclature is based on the level ordering at t=0 and will be constantly used even if the order of the levels changes during the simulation.

**Figure 2:**
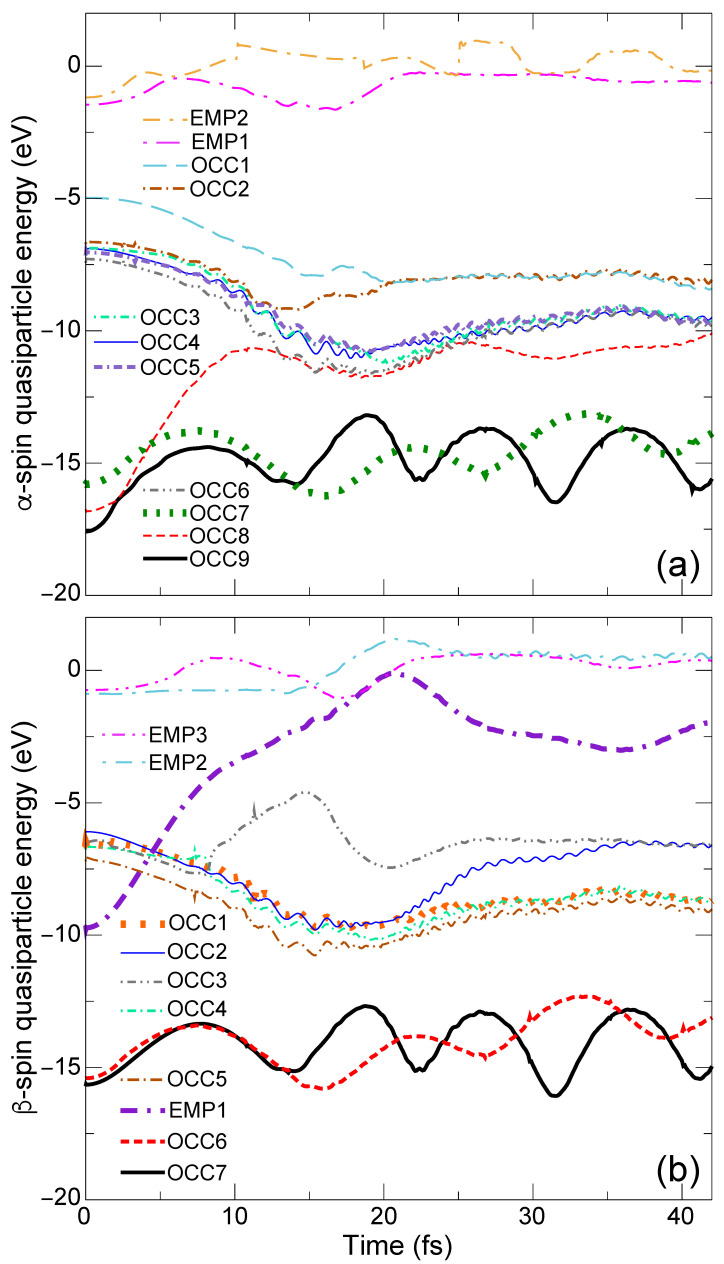
Time evolution of (**a**) α-spin and (**b**) β-spin QP energy eigenvalues $\varepsilon_{n}^{\mathrm{QP}}\left(R\left(t\right)\right)$.

**Figure 3:**
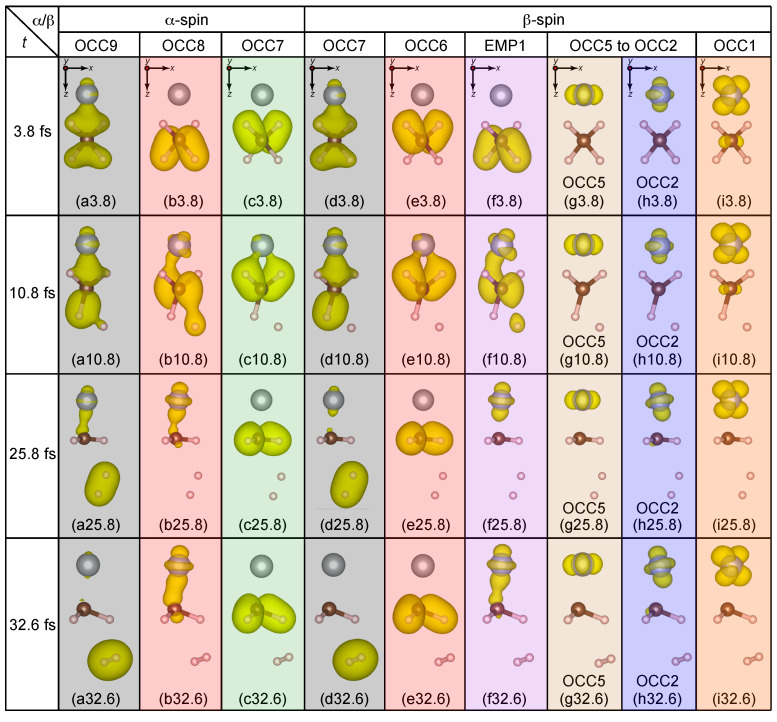
Panels (**a**–**i**) represent the charge densities of the levels of interest for the H ejection opposite to the Ni side for each time instant at t=3.8, 10.8, 25.8, and 32.6 fs. The isosurface for the charge density plots was set at $1\times 10^{-8}$ electrons/${Å}^{3}$. The directions of viewing are indicated for reference in the uppermost panels (**a**).

The time evolution of the QP energy eigenvalues $\varepsilon_{n}^{\mathrm{QP}}\left(R\left(t\right)\right)$ of the α- and β-spin levels is shown, respectively, in Figure 2a,b, while, the charge densities of the different levels of interest are shown at t=3.8, 10.8, 25.8, and 32.6 fs in a tabular format below the QP energy plots in Figure 3.

The levels of interest are (a) OCC9 (GS 12th HOMO−8 level), (b) OCC8 (GS 13th HOMO−7 level), (c) OCC7 (GS 14th HOMO−6 level) for α-spin, and (d) OCC7 (GS 12th HOMO−6 level), (e) OCC6 (GS 13th HOMO−5 level), (f) EMP1 (GS 14th HOMO−4 level), (g) OCC5 (GS 15th HOMO−3 level), (h) OCC2 (GS 18th HOMO level), (i) OCC1 (GS 19th LUMO level) for β-spin. The alphabets a-i followed by the simulation time in femtoseconds (fs) are used to represent the charge density panels.

At t=0, the QP energies of the β-spin OCC1 and EMP1 levels are, respectively, $\varepsilon_{{\mathrm{OCC}1}_{\beta }}=-6.7$ eV [thick orange dotted line in Figure 2b] and $\varepsilon_{{\mathrm{EMP}1}_{\beta }}=-9.6$ eV [thick violet dashed-dotted-dashed line in Figure 2b]. They are the Ni 3d orbital and the CH_4_ bonding orbital as seen in Figure 1h,e, respectively. According to EQPT, $\varepsilon_{\mathrm{HOMO}-4_{\beta }}=E_{\mathrm{GS}}-E_{\mathrm{HOMO}-4_{\beta }}$ and $\varepsilon_{{\mathrm{OCC}1}_{\beta }}=E_{\mathrm{photo}}-E_{\mathrm{HOMO}-4_{\beta }}$, where $E_{\mathrm{GS}}$, $E_{\mathrm{photo}}$, and $E_{\mathrm{HOMO}-4_{\beta }}$ are the total energies of the GS, the photoexcited state, and the (N−1)-electron state with one electron missing at the the 14th ${\mathrm{HOMO-4}}_{\beta }$ level, respectively. Note that $\varepsilon_{\mathrm{HOMO}-4_{\beta }}$ is equal to −IP of the methane fragment in the CH_4_ + Ni system. Similarly, EA $=E_{\mathrm{GS}}-E_{\mathrm{anion}}$ and $\varepsilon_{{\mathrm{EMP}1}_{\beta }}=E_{\mathrm{anion}}-E_{\mathrm{photo}}$, where $E_{\mathrm{anion}}$ is the total energy of the anionic state with one electron added to the LUMO level of the GS. From these relations, the photoabsorption energy (PAE) for this excitation $E_{\mathrm{photo}}-E_{\mathrm{GS}}$ can be obtained in two different ways, $\varepsilon_{{\mathrm{OCC}1}_{\beta }}-\varepsilon_{{\mathrm{HOMO}-4}_{\beta }}=-6.7-\left(-14.9\right)$ eV = 8.2 eV and −EA $-\;\varepsilon_{{\mathrm{EMP}1}_{\beta }}=-1.0-\left(-9.6\right)$ eV = 8.6 eV. The similarity in these two values clearly indicates the accuracy of our calculation. The resulting PAE (the averaged value is 8.4 eV) is about 1.8 eV lower than that for pristine CH_4_ (10.2 eV [5,6,7]).

At t=3.8 fs, we see that the charge densities of all QP levels [Figure 3a–i3.8] do not change significantly from those of the GS [Figure 1a–h], with the exception that the ${\mathrm{OCC8}}_{\alpha }$ and ${\mathrm{OCC7}}_{\alpha }$ levels for the ES reference [Figure 3b,c3.8] are the swapped ${\mathrm{HOMO-7}}_{\alpha }$ [Figure 1b] and ${\mathrm{HOMO-6}}_{\alpha }$ (not shown in Figure 1) levels for the GS reference. On the other hand for 4≤t<5 fs, the ${\mathrm{EMP1}}_{\beta }$ level [thick violet dashed-dotted-dashed line in Figure 2b] crosses with the other ${\mathrm{OCC5}}_{\beta }{\mathrm{-OCC1}}_{\beta }$ levels [OCC5 – brown dotted-dashed line, OCC4, light-green dotted-dashed line, OCC3, gray dotted-dashed-dotted line, OCC2, thin blue solid line, and OCC1, thick orange dotted line in Figure 2b]. The charge density of ${\mathrm{EMP1}}_{\beta }$ at t=3.8 fs [Figure 3f3.8] is localized on the methane fragment but its population is shifted to the Ni side at t=10.8 fs [Figure 3f10.8]. On the other hand, the charge density of OCC1 at t=3.8 and 10.8 fs [Figure 3i3.8,10.8] does not change and continues to exhibit a Ni 3d character. Therefore, the electronic structure (mainly composed of the occupied orbitals) does not deviate significantly from the BO surface during level crossings, and the breaking acceleration does not exceed the SH threshold value $\delta_{\mathrm{clone}}$. Consequently, the simulation is continued without SH. As the simulation progresses, two H atoms opposite the Ni side (H1 and H2 in Figure 1) begin to dissociate from the CH_4_ fragment of the CH_4_ + Ni system.

Interesting parallels emerge in both the α- and β-spin QP energies in Figure 2a,b, respectively. While the QP energies of the ${\mathrm{OCC8}}_{\alpha }$ [red dashed line in Figure 2a] and the ${\mathrm{EMP1}}_{\beta }$ [thick violet dashed-dotted-dashed line in Figure 2b] levels increase (∼−17 eV to ∼−10 eV for ${\mathrm{OCC8}}_{\alpha }$ and −9.6 eV to ∼−2 eV for ${\mathrm{EMP1}}_{\beta }$) with time, the QP energies of the ${\mathrm{OCC7}}_{\alpha }$ [thick green dotted line in Figure 2a] and ${\mathrm{OCC6}}_{\beta }$ [thick red dashed line in Figure 2b] levels oscillate analogously around ∼−15 eV. The resemblances in the temporal evolution of ${\mathrm{OCC9}}_{\alpha }$ and ${\mathrm{OCC7}}_{\beta }$, ${\mathrm{OCC8}}_{\alpha }$ and ${\mathrm{EMP1}}_{\beta }$, and ${\mathrm{OCC7}}_{\alpha }$ and ${\mathrm{OCC6}}_{\beta }$ indicate that these orbitals are spin-paired with each other. This is further reflected in the similarities in the charge densities in Figure 3a3.8–32.6,d3.8–32.6 for ${\mathrm{OCC9}}_{\alpha }$ and ${\mathrm{OCC7}}_{\beta }$, Figure 3b3.8–32.6,f3.8–32.6for ${\mathrm{OCC8}}_{\alpha }$ and ${\mathrm{EMP1}}_{\beta }$, and Figure 3c3.8–32.6,e3.8–32.6 for ${\mathrm{OCC7}}_{\alpha }$ and ${\mathrm{OCC6}}_{\beta }$ throughout the entire duration (t=3.8, 10.8, 25.8, and 32.6 fs) of the simulation.

The charge population in both ${\mathrm{OCC9}}_{\alpha }$ and ${\mathrm{OCC7}}_{\beta }$ is initially spread toward the Ni side (charge densities for t=3.8,10.8 fs) before being shifted towards the two ejected H atoms (H1 and H2) at t=25.8 fs [Figure 3a,d25.8]. Subsequently, the H1 and H2 atoms approach each other and begin to form a H−H bond. Finally, at t=32.6 fs, these α- and β-spin levels become the completely isolated σ orbital of H−H [Figure 3a,d32.6]. At this time, the H_1_−H_2_ bond length is 0.72Å, which is very close to the experimental H_2_ bond length, 0.74Å [46], and the bond distances of C−H_1_ and C−H_2_ are 2.51Å and 2.78Å, respectively, which are both considerably large. We additionally perform a contour analysis of the charge densities shown in Figure 3a,d32.6 to obtain quantitative estimates of charge populations around the H_2_ fragment [Supplementary Information Figure S1a]. We compare this with the charge contour of an isolated H_2_ molecule obtained from a single-point LDA calculation [Supplementary Information Figure S1b]. We find an extremely good agreement between the TDGW-MD and the LDA results demonstrating the computational reliability of our predicted dynamics using TDGW-MD. Moreover, the QP energies of the ${\mathrm{OCC9}}_{\alpha }$ and ${\mathrm{OCC7}}_{\beta }$ levels at t=32.6 fs in Figure 2a,b are, respectively, −15.8 eV and −15.3 eV, which are also very close to the −IP of the hydrogen molecule 15.4 eV [47]. Therefore, we conclude that an isolated hydrogen molecule $H_{2}$ is created as a product of this photolysis reaction in this very short time period. Based on this trajectory, we speculate that the photolysis reaction would be CH_4_ + Ni $\overset{\hbar \omega =8.4\mathrm{eV}}{\to }$ CH_2_−Ni + H_2_.

### 3.2. H Ejection Towards the Ni Side

**Figure 4:**
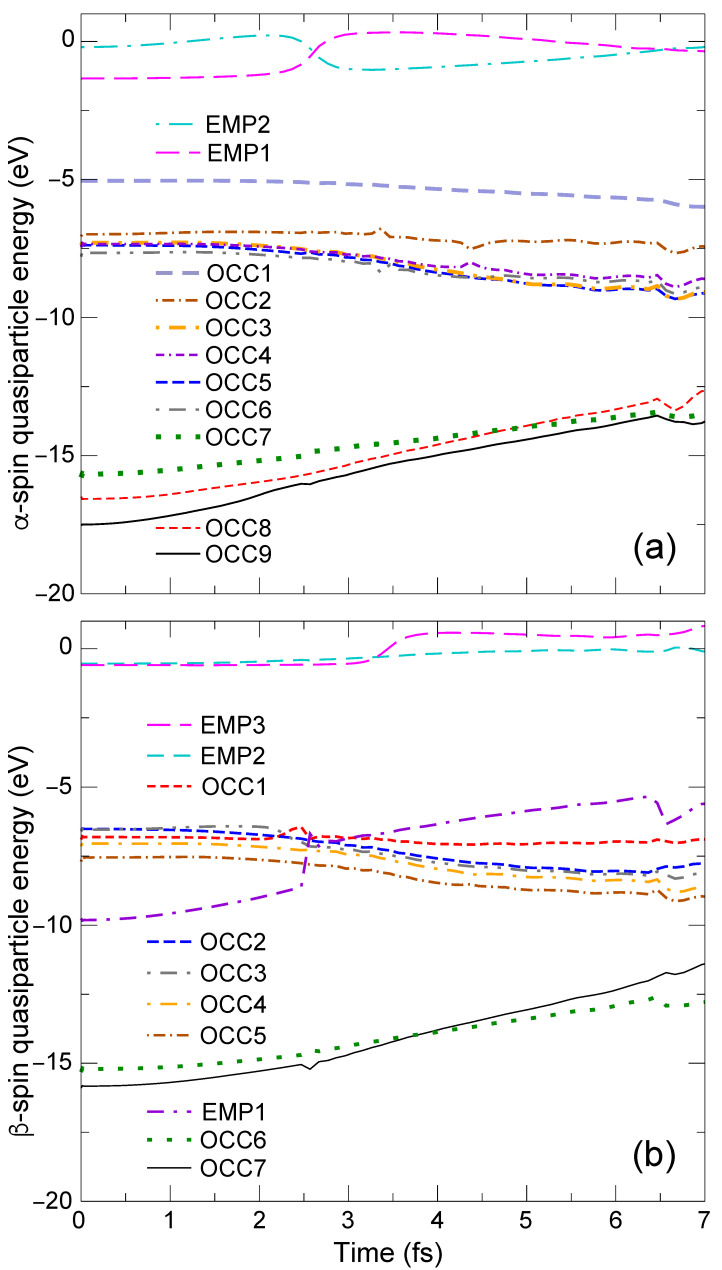
Early time behavior of (**a**) α-spin, (**b**) β-spin QP energy eigenvalues $\varepsilon_{n}^{\mathrm{QP}}\left(R\left(t\right)\right)$ for the H ejection towards the Ni side.

**Figure 5:**
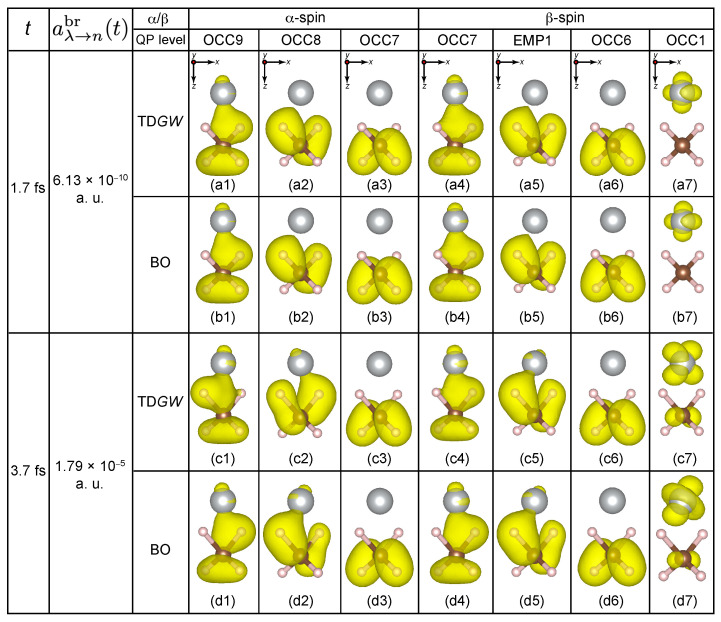
Comparison of TDGW charge densities (**a**,**c**) with BO charge densities (**b**,**d**) for the ES reference at t=1.7 fs (a1–7) and (b1–7) and t=3.7fs (c1–7) and (d1–7). The isosurface for the charge density plots was set at $1\times 10^{-8}$ electrons/${Å}^{3}$. The directions of viewing are indicated for reference in the uppermost panels (**a**).

We excite an electron from the slightly deeper ${\mathrm{HOMO-1}}_{\beta }$ level of ${\mathrm{CH}}_{4}$, which is the 13th ${\mathrm{HOMO-5}}_{\beta }$ level of the CH_4_ + Ni system, to the 19th empty Ni $3d{\mathrm{LUMO}}_{\beta }$ level. The original ${\mathrm{HOMO-5}}_{\beta }$ is now empty and is called “${\mathrm{EMP1}}_{\beta }$”, while the original ${\mathrm{LUMO}}_{\beta }$ is now occupied and is called “${\mathrm{OCC1}}_{\beta }$”. Figure 4a,b shows the early time behavior of the QP energy eigenvalues $\varepsilon_{n}^{\mathrm{QP}}\left(R\left(t\right)\right)$ of the α- and β-spin levels, respectively. The TDGW and the BO charge densities for the ES reference at t=1.7 fs and t=3.7 fs are shown in Figure 5.

At t=0, the QP energy of ${\mathrm{EMP1}}_{\beta }$ [violet dashed-dotted-dashed line in Figure 4b] is $\varepsilon_{{\mathrm{EMP}1}_{\beta }}=-9.8$ eV and the QP energy of ${\mathrm{OCC1}}_{\beta }$ [red dashed line in Figure 4b] is $\varepsilon_{{\mathrm{OCC}1}_{\beta }}=-6.9$ eV. They are the CH_4_ bonding orbital and the Ni 3d orbital as seen in the charge density plots in Figure 5a5,7 at t=1.7 fs. From EQPT, the required energy for this photoexcitation can be calculated as either the difference between $\varepsilon_{{\mathrm{OCC}1}_{\beta }}$ of this photoexcited state and $\varepsilon_{{\mathrm{HOMO}-5}_{\beta }}$ of the GS, or the difference between $\varepsilon_{{\mathrm{LUMO}}_{\beta }}$ of the GS state, i.e., −EA, and $\varepsilon_{{\mathrm{EMP}1}_{\beta }}$ of this photoexcited state. The former is −6.9−(−15.1) eV = 8.2 eV and the latter is −1.0−(−9.8) eV = 8.8 eV, which are close to each other. This again demonstrates the accuracy of our simulation. The resulting PAE (the averaged value is 8.5 eV) is about 1.7 eV lower than that for pristine CH_4_ (10.2 eV [5,6,7]).

At t=1.7 fs, the TDGW and BO charge densities are quite similar to each other as seen in Figure 5a1–7,b1–7. They also resemble the GS charge densities in Figure 1a–h. This indicates that the non-adiabatic effect is very weak at this time. Indeed, the breaking acceleration $a_{\lambda \to n}^{\mathrm{br}}=6.13\times 10^{-10}$ a.u. is much smaller than the threshold value $\delta_{\mathrm{clone}}=3\times 10^{-6}$ a.u. In the 2.5≤t≤3.0 fs time interval, we see level crossings between the ${\mathrm{EMP1}}_{\beta }$ level [violet dashed-dotted-dashed line in Figure 4b] and the other ${\mathrm{OCC5}}_{\beta }{\mathrm{-OCC1}}_{\beta }$ levels [OCC5, brown dotted-dashed line, OCC4, orange dotted-dashed line, OCC3, gray dotted-dashed-dotted line, OCC2, blue dashed line, and OCC1, red dashed line in Figure 4b]. In contrast, there is no such level crossing in the lower α-spin levels besides that between the EMP1 [pink dashed line in Figure 4a] and EMP2 [light blue dotted-dashed line in Figure 4a] levels at t=2.6 fs.

At t=3.7 fs, the breaking acceleration $a_{\lambda \to n}^{\mathrm{br}}=1.79\times 10^{-5}$ a.u. exceeds the threshold value $\delta_{\mathrm{clone}}=3\times 10^{-6}$ a.u., similar to the case of pristine methane [24]. Indeed, the TDGW charge densities at this time instant [Figure 5c1–7] are somewhat different from the BO charge densities with the same geometry [Figure 5d1–7]. For example, those of the ${\mathrm{OCC9}}_{\alpha }$ and ${\mathrm{OCC8}}_{\alpha }$ levels are different as if the left and right charge lobes were reversed. Moreover, those of the ${\mathrm{OCC1}}_{\beta }$ level are slightly different in terms of the Ni 3d character. Therefore, the simulation undergoes SH at t=3.7 fs. In our Ehrenfest framework, we perform SH to the BO surface for the GS reference.

**Figure 6:**
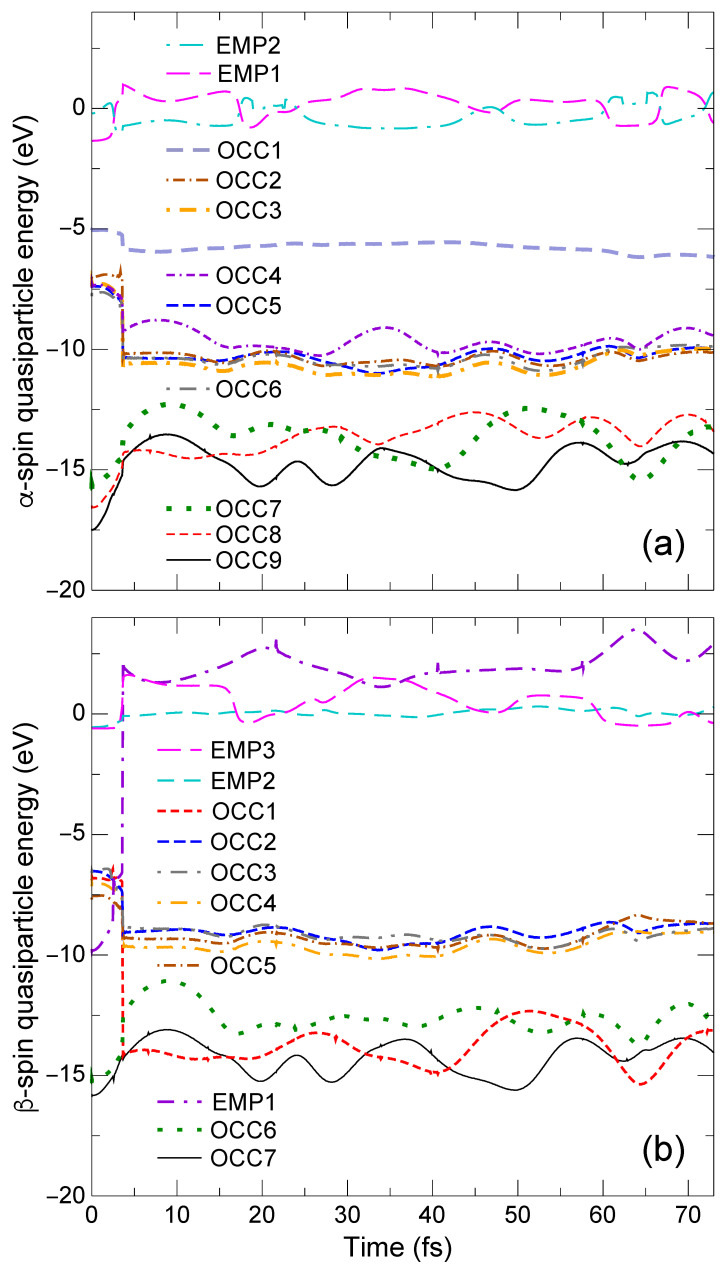
Time evolution of (**a**) α-spin, (**b**) β-spin QP energy eigenvalues $\varepsilon_{n}^{\mathrm{QP}}\left(R\left(t\right)\right)$.

**Figure 7:**
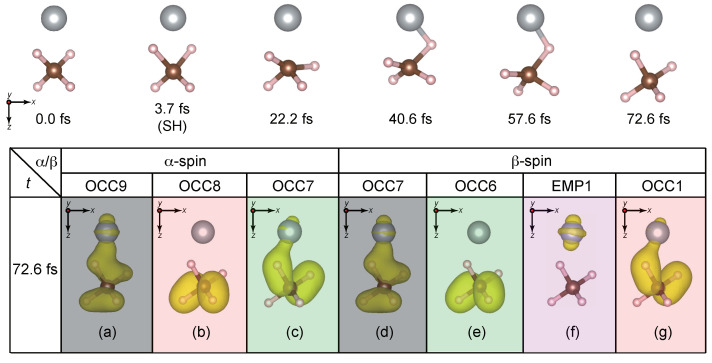
Time evolution of atomic geometry for the H ejection in the Ni side. Panels (**a**–**g**) represent charge densities of relevant QP levels at t=72.6 fs. The isosurface for the charge density plots was set at $1\times 10^{-8}$ electrons/${Å}^{3}$. The directions of viewing are indicated for reference.

The time evolution of the QP energies (for the simulation with SH at t=3.7 fs) are shown in Figure 6a,b, respectively, for α- and β-spins while the trajectory of this simulation is presented in Figure 7.

At the SH time, ${\mathrm{EMP1}}_{\beta }$ level [violet dashed-dotted-dashed line in Figure 6b] jumps up above the vacuum level, while ${\mathrm{OCC1}}_{\beta }$ level [red dashed line in Figure 6b] falls down to ∼−14 eV. Around this SH time, the two H atoms in the Ni side (H3 and H4 in Figure 1) start to move away from the C atom and approach the Ni atom. Thereafter, ${\mathrm{OCC9}}_{\alpha }{\mathrm{-OCC7}}_{\alpha }$, ${\mathrm{OCC7}}_{\beta }$, ${\mathrm{OCC6}}_{\beta }$ and ${\mathrm{OCC1}}_{\beta }$ levels oscillate in their corresponding QP energies around −14 eV [Figure 6a,b]. The similarity in their temporal variation is reflected in the charge densities shown in Figure 7a–e,g, implying that these orbitals are spin-paired with each other.

With time, the H3 and H4 atoms begin to move away from the Ni atom. At t=22.2 fs, H3 and H4 return to the methane side, but around the [40.6, 57.6] fs time interval, H3 is bound to the Ni atom. However, this bonding is not strong with the H_3_−Ni bond being broken, and a subsequent return of H3 toward methane. Finally, at t=72.6 fs, the original geometry is virtually retrieved. Indeed, the charge densities at t=72.6 shown in Figure 7a–g are very close to those of the GS charge densities in Figure 1. At the same time, interestingly, the Ni atom starts to move away from the CH_4_ fragment which is clearly evident at t=72.6 fs from the C−Ni distance around 2.29Å. From Figure 6a,b around t=72.6 fs, we see that the QP energies $\varepsilon_{{\mathrm{OCC}9}_{\alpha }}$-$\varepsilon_{{\mathrm{OCC}7}_{\alpha }}$, $\varepsilon_{{\mathrm{OCC}7}_{\beta }}$, $\varepsilon_{{\mathrm{OCC}6}_{\beta }}$, and $\varepsilon_{{\mathrm{OCC}1}_{\beta }}$ oscillate in their QP energies around the mean value of −14 eV, which is very close to − IP =−13.7±0.5 eV of the pristine methane [24]. (Experimental IP is 12.6–14.8 eV [43,44,45].) In addition, all empty levels (EMP1, EMP2, etc.) are above the vacuum level or just close to the vacuum level regardless of the spin, as seen in Figure 6a,b, reflecting the negative EA of the pristine methane molecule. All these observations indicate that the combined CH_4_−Ni system is undergoing dissociation into the CH_4_ and Ni fragments, i.e, CH_4_ + Ni → CH_4_−Ni $\overset{\hbar \omega =8.4\mathrm{eV}}{\to }$ CH_4_ + Ni.

## 4. Discussion

We have considered two possible pathways of the photolysis of methane in the presence of a Ni atom depending on either the β-spin 14th (${\mathrm{HOMO-4}}_{\beta }$) level (HOMO of the methane fragment) or the 13th (${\mathrm{HOMO-5}}_{\beta }$) level (HOMO−1 of the methane fragment) being excited to the 19th (${\mathrm{LUMO}}_{\beta }$) level. In both the cases, we estimated the required PAE in two different ways under EQPT. One approach is $\varepsilon_{{\mathrm{OCC}1}_{\beta }}$ of the ES minus $\varepsilon_{\mathrm{HOMO}-4_{\beta }}$ (or $\varepsilon_{\mathrm{HOMO}-5_{\beta }}$) of the GS and the other is −EA minus $\varepsilon_{{\mathrm{EMP}1}_{\beta }}$ of the ES. (IP of the methane fragment is $-\varepsilon_{\mathrm{HOMO}-4_{\beta }}$.) The resulting PAEs via either approach are close to each other, suggesting the accuracy of the present calculation. The PAEs are 8.4 eV and 8.5 eV, respectively, for the 14th and the 13th level excitations which are lower than that of 10.2 eV for pristine methane, indicating the reduction in the PAE due to the existence of a Ni atom. This reduction in PAE is due to the excitation of an electron from a C 2p orbital [Figure 1d,e] to an intermediate 3d orbital of Ni [Figure 1h] instead of to the higher energy 3s orbital of C (in the pristine CH_4_ case). As a matter of fact, photolysis of methane on a Pt(111) surface has experimentally been shown to occur at an excitation energy of around 6.4 eV (∼193 nm) [48], which is significantly lower than 10.2 eV (∼122 nm) in the absence of such a substrate further exemplifying the crucial role played by transition metal atoms in making photochemical reactions more accessible.

It is noteworthy that, in both pathways, two hydrogen atoms are simultaneously ejected from the methane molecule at the beginning, which is a distinct characteristic of the present results. On the contrary, in the pristine methane case, only one H atom is ejected by a single photon absorption. Why are two H atoms ejected simultaneously from CH_4_ despite undergoing an excitation via a single photon absorption in the presence of a Ni atom? The reason for this is that the C−H bonds in the CH_4_ molecule become considerably weakened while the C−Ni bonding becomes stronger via *p*-*d* orbital hybridization.

When an electron is excited from the 14th level to the 19th level, two H atoms opposite Ni are ejected from the methane molecule, and they eventually combine to become an isolated hydrogen molecule. The QP energy of the HOMO level of this ejected hydrogen molecule is ∼−15.5 eV irrespective of the spin, which is close to the −IP of the isolated hydrogen molecule 15.4 eV [47]. The QP wavefunction corresponding to the ejected hydrogen molecule is completely localized at the hydrogen molecule, and there is no charge fraction remaining in the CH_2_−Ni side (see also Supplementary Information (SI) for the contour plots of the charge density). Thus, we can conclude that a hydrogen molecule is produced as a product. This is a sensational result because two hydrogen atoms are ejected from one methane molecule by a single photon absorption and they automatically combine to become a hydrogen molecule. This clearly demonstrates the important role played by Ni in providing a more efficient route for photolysis. This pathway leads to the formation of the H_2_ molecule as there does not exist an ‘absorber’ such as Ni to impede the motion of the two H atoms. This reaction is closely related to the thermocatalytic or solar-aided decomposition of methane, where a transition metal atom or cluster mediates the growth of nanocarbon materials together with the production of hydrogen molecules. For example, there is a possibility that, if two methane molecules are attached to a Ni cluster, the remaining C atoms can combine to produce the C_2_ dimer subsequent to several photolysis reactions that may result in the dissociation of four hydrogen molecules. With the addition of more methane molecules, this process may be continued to produce carbon nanomaterials.

In contrast, when two hydrogen atoms facing the Ni atom are ejected, the ejected H atoms cannot escape because of the strong attractive forces exerted by both the Ni and methylene (CH_2_) fragments. The two H atoms initially approach the Ni atom due to the inertia of motion but eventually return to the CH_4_ fragment. There are three competing factors that govern the entire dynamic: the inertia of motion of the ejected H, the Coulombic interaction between the H and Ni atoms, and the covalent interaction between the H and C atoms. During the initial stage of the photolysis, as the ejected H atoms approach the Ni atom, their inertia of motion carries them away from the C atom; see Figure 7. After SH to the GS-BO surface, the strength of the covalent interaction exceeds this inertia and forces the ejected H atoms to return. With this return, the inertia of motion in the reverse direction increases leading to the H atoms overshooting their original positions at t=22.2 fs. In this process, the overall CH_4_ geometry strongly deviates from its stable configuration, which initiates the rebound of one of the H atoms toward Ni. Mediated by a weak Coulomb interaction as a result of charge transfer from this H atom to the Ni atom, this H temporarily bonds with Ni around t=40.6–57.6 fs. However, the influence of the C atom continues to persist, leading to the return of this ejected H to the original position at t=72.6 fs [Figure 7a–g]. Because of these three competing factors, none of the H atoms can get dissociated after ejection, but rather prefer to return to (nearly) recover the original methane geometry. At the same time, during the reversal of the H atom from Ni towards CH_4_, the increase in the inertia of H in the opposite direction initiates the concerted motion of CH_4_ away from Ni. This can be seen not only from the atomic trajectory but also from the fact that methane-derived QP levels have energy values similar to −IP of pristine methane. Therefore, in this case, we conclude that the combined CH_4_−Ni system is dissociated into CH_4_ and Ni.

We next comment on the reliability of our simulation results despite the short MD simulation times. Photolysis reactions are generally ultrafast [49] as they usually complete within several tens of femtoseconds. Therefore, even though the simulation times seem to be short in our work, the match between QP energies and experimental values for both the trajectories is clearly evident. Therefore, we argue that the present results are reliable.

In addition to the reliability and accuracy of our results, these were obtained in reasonable wall clock times. The simulation of H ejection away from/toward Ni took 6/10 days using four nodes with 48 MPI processes on the Numerical Materials Simulator supercomputer at the National Institute for Materials Science.

Through our simulations, we show how two H atoms can be dissociated via a single photon absorption eventually leading to the formation of an isolated H_2_ molecule. This is achieved at a much lower PAE of 8.4 eV, compared to pristine methane (10.2 eV). Overall, our work presents an opportunity for experimental verification of our observations. The results obtained from our simulations are accurate as they are obtained without any adjustable/fitting parameters and are based on sound mathematical principles.

## Figures and Tables

**Table 1:** The calculated QP energies (in units of eV) within the GWA for the GS reference using the initial geometry. The 14th level (14*) corresponds to the CH_4_ HOMO.

Level	α-Spin (eV)		β-Spin (eV)	
21	LUMO	−1.0	LUMO+2	−0.3
20	HOMO	−6.9	LUMO+1	−0.8
19	HOMO−1	−9.9	LUMO	−1.0
18	HOMO−2	−10.3	HOMO	−8.9
17	HOMO−3	−10.4	HOMO−1	−9.5
16	HOMO−4	−10.6	HOMO−2	−9.3
15	HOMO−5	−10.6	HOMO−3	−9.4
14*	HOMO−6	−15.0	HOMO−4	−14.9
13	HOMO−7	−15.7	HOMO−5	−15.1
12	HOMO−8	−16.0	HOMO−6	−15.8
11	HOMO−9	−23.8	HOMO−7	−23.6

## Data Availability

The data that support the findings of this study are available within the article and its supplementary files, away_from_Ni_MD.xyz and towards_Ni_MD.xyz. The xyz files can be visualized using the VMD software package [50]. The TOMBO executable used for performing NA-ES-TDGW-MD simulations and one-shot GW calculations is available upon request.

## Supplementary Materials

The following supporting information can be downloaded at: https://www.mdpi.com/article/10.3390/nano14221775/s1, Figure S1: Contour plots of the $H_{2}$ charge density; MD Trajctory S1: away_from_Ni_MD.xyz; MD Trajectory S2: towards_Ni_MD.xyz; see Data Availability Statement to view these .xyz files.

## Abbreviations

The following abbreviations are used in this manuscript:

DFT	density functional theory
TD	time-dependent
TDDFT	time-dependent density functional theory
LDA	local density approximation
ALDA	adiabatic local density approximation
MD	molecular dynamics
ES	excited state
GS	ground state
QP	quasiparticle
EQPT	extended quasiparticle theory
vac	vacuum level
GWA	GW approximation
SH	surface hopping
TDGW	Non-adiabatic excited-state time-dependent GW
PW	plane wave
AO	atomic orbital
HOMO	highest occupied molecular orbital
LUMO	lowest unoccupied molecular orbital
IP	ionization potential
EA	electron affinity
PAE	photoabsorption energy
