# Supplementary material for: Non-Adiabatic Excited-State Time-Dependent GW (TDGW) Molecular Dynamics Simulation of Nickel-Atom Aided Photolysis of Methane to Produce a Hydrogen Molecule
Source: Nanomaterials (Basel). 2024 Nov 5;14(22):1775. doi: 10.3390/nano14221775 (PMC11597270; doi:10.3390/nano14221775)
Supplement: Supplementary file 1 [file nanomaterials-14-01775-s001.zip › nanomaterials-3262509-Supplementary.pdf]

Supplementary Information

Non-adiabatic excited-state time-dependent GW (TDGW) molecular dynamics simulation of nickel-atom aided photolysis of methane to produce a hydrogen molecule

Aaditya Manjanath [1], Ryoji Sahara [1], Yoshiyuki Kawazoe [2,3,4], and Kaoru Ohno [1,5,*]

[1] Research Center for Structural Materials, National Institute for Materials Science (NIMS), 1-2-1 Sengen, Tsukuba, Ibaraki, 305-0047, Japan
[2] New Industry Creation Hatchery Center, Tohoku University, 6-6-4 Aramaki Aza Aoba, Aoba-ku, Sendai, Miyagi, 980-8579 Japan
[3] School of Physics, Institute of Science, Suranaree University of Technology, 111 University Avenue, Nakhon Ratchasima, 30000 Thailand
[4] Physics and Nanotechnoloy, SRM Institute of Science and Technology, Kattankurathur, Tamil Nadu, 603203 India
[5] Department of Physics, Graduate School of Engineering, Yokohama National University (YNU), 79-5 Tokiwadai, Hodogaya-ku, Yokohama 240-8501, Japan; ohno@ynu.ac.jp

We demonstrated that the H ejection away from the Ni side facilitates the formation of a hydrogen molecule, i.e., $CH_4 + Ni \xrightarrow{\hbar\omega} CH_2{-}Ni + H_2$, with the quasiparticle level corresponding to it having the energy close to the negative ionization potential of an isolated H_2 molecule. We additionally perform a contour analysis of the charge densities shown in Figs. 3(a32.6) and (d32.6) in the main text to obtain quantitative estimates of charge populations around the H_2 fragment [Fig. S1(a)]. We compare this with the charge contour of an isolated H_2 molecule obtained from a single-point LDA calculation [Fig. S1(b)]. We find an extremely good agreement between the TDGW-MD and the LDA results indicating that a H_2 molecule is produced for the H away from Ni trajectory within a short timescale.

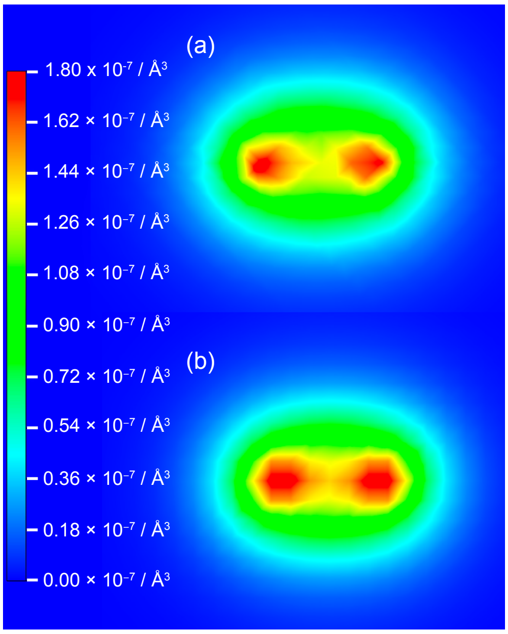

Figure S1: Contour plots of the H_2 charge density obtained from (a) the on-the-fly TDGW-MD result for the $CH_4{-}Ni$ system at $t = 32.6$ fs [Fig. 3(d32.6) in the main text] and (b) the self-consistent LDA result for the purely isolated H_2 system.
